# ﻿One new species and two new records of Pyrrhocoridae (Hemiptera, Heteroptera) from China

**DOI:** 10.3897/zookeys.1210.125457

**Published:** 2024-08-27

**Authors:** Ping Zhao, Minmin Ou, Liangming Cao, Huaiyu Liu, Jianyun Wang

**Affiliations:** 1 Key Laboratory of Environment Change and Resources Use in Beibu Gulf of Ministry of Education, Nanning Normal University, Nanning 530001, China Nanning Normal University Nanning China; 2 Key Laboratory of Forest Protection of National Forestry and Grassland Administration, Ecology and Nature Conservation Institute, Chinese Academy of Forestry, Beijing 100091, China Ecology and Nature Conservation Institute, Chinese Academy of Forestry Beijing China; 3 Department of Entomology and MOA Key Lab of Pest Monitoring and Green Management, College of Plant Protection, China Agricultural University, Beijing 100193, China China Agricultural University Beijing China; 4 Environment and Plant Protection Institute, Chinese Academy of Tropical Agricultural Sciences, Haikou 571101, China Environment and Plant Protection Institute, Chinese Academy of Tropical Agricultural Sciences Haikou China

**Keywords:** Oriental region, red bugs, South China, taxonomy, tropical zone

## Abstract

A new species, *Dindymusalbonotum* Zhao & Cao, **sp. nov.**, and two newly recorded species, *Euscopusrobustus* Stehlík, 2005 and Brancucciana (Rubriascopus) orientalis Stehlík & Jindra, 2008, belonging to the family Pyrrhocoridae Amyot & Serville, 1843 (Hemiptera, Heteroptera, Pyrrhocoroidea) from China are described and illustrated.

## ﻿Introduction

The currently known pyrrhocorid fauna (Hemiptera, Heteroptera, Pyrrhocoroidea) of China includes 15 genera and 40 species (Zhao et al. 2022). Distant (1903) was the first to comprehensively study the pyrrhocorids of the Oriental region and reported 52 species, including 20 from China. Between 1929 and 2022, there has been a gradual accumulation of taxonomic research on the Pyrrhocoridae of China, including works by Hussey (1929), Blöte (1931), Hsiao (1964), Liu (1981a), Stehlík and Kerzhner (1999), Kerzhner (2001), Stehlík and Jindra (2006a, 2006b), Rédei et al. (2009), and Zhao et al. (2022). Despite this, the number of pyrrhocorid species documented in China is less than 10% of the worldwide total of 525 species (49 genera) (Zhao et al. 2022). Considering the diversity of habitats across China, including the species-rich Oriental portion, it is likely that the Chinese pyrrhocorid fauna has not yet been fully documented.

In this paper we describe a pyrrhocorid species new to science from the Oriental part of China: *Dindymusalbonotum* sp. nov. We also report the first distribution records for Brancucciana (Rubriascopus) orientalis Stehlík & Jindra, 2008 and *Euscopusrobustus* Stehlík, 2005 in China.

## ﻿Materials and methods

All studied materials are deposited in the Entomological Museum of China Agricultural University (CAU), Beijing, China. External structures were examined using a binocular dissecting microscope. Species descriptions were based on naturally dried specimens. Male genitalia were put into a 1.50 ml centrifuge tube and soaked in pure lactic acid heated to 80 °C in constant temperature metal bath for ~15 min to remove soft tissue, then rinsed in boiling distilled water for ~1 min and dissected under a microscope. Dissected parts of the genital structures were placed in a plastic microvial with lactic acid under each corresponding specimen. Habitus photographs of all the species were taken with a Canon D60 SLR camera (Canon Inc., Tokyo, Japan). Male external genitalia were photographed with the aid of a Nikon SMZ25 stereomicroscope (Nikon Corporation, Tokyo, Japan). Measurements were obtained using a calibrated micrometer. All measurements are presented in millimeters (mm). The classification system mainly follows that of Rédei et al. (2009), Aukema et al. (2013), Stehlík and Kerzhner (1999), and Zhao et al. (2022). The morphological terminology of male genitalia follows Schaefer (1977) and Ahmad and Zaidi (1986); the terminology of the female genitalia is according to the paper published by Zhou and Rédei (2020). The generic and specific names in the text are arranged alphabetically.

## ﻿Taxonomy

**Family Pyrrhocoridae Amyot & Serville, 1843**

### ﻿Genus Brancucciana

Taxon classificationAnimaliaHemipteraPyrrhocoridae

Ahmad & Zaidi, 1986

C1FE2B7A-643F-5B20-B7BE-A030BEB0A744

Brancucciana Ahmad & Zaidi, 1986: 423; Stehlík 2007: 109; Aukema et al. 2013: 402. Type species by original designation: Brancuccianabhutanensis Ahmad & Zaidi, 1986.
Ascopus Hsiao, 1964: 402, 405 (junior homonym of Ascopus Marshall, 1951, Coleoptera). Type species by original designation: Ascopusrufa Hsiao, 1964: 403, 405.
Ascopocoris Stehlík & Kerzhner, 1999: 123; Stehlík and Jindra 2006a: 61. Unnecessary new name for Ascopus Hsiao, 1964.

#### Generic character.

Body generally sanguineous, broadly oval. Head as long as broad and nearly triangular, apical part anteriorly produced; eyes smaller and bulging, posterior margin of eyes adjacent to anterior margin of pronotum; antennae shorter and subequal in length to half of body; first antennal segment subequal to head length, apical half dilated; labium reaching to hind coxae, second segment longest, fourth shortest, first subequal in length to third. Length of pronotum distinctly shorter than its width, lateral pronotal margins reflexed; scutellum equilateral triangular; metathoracic scent gland ostiole large; membrane of hemelytron reaching just beyond the apex of abdomen, costal margin of corium reflexed. Sterna of thorax centrally with longitudinal ridge. Femora thickened, ventral side of subapcial part of fore femora with several dentate processes; third segment of tarsus of hind leg longer than first and second segments together. Pygophore somewhat rounded, ventroposterior margin medially with a knob; paramere F-shaped; aedeagus with both thecal and conjunctival appendages.

#### Distribution.

Oriental region.

#### Remark.

Hsiao (1964) established the genus *Ascopus* Hsiao, 1964 with *Ascopusrufa* Hsiao, 1964 as type species. Liu (1987) found a second species, *Ascopussinuaticollis* Liu, 1987. However, the genus name, *Ascopus* was already preoccupied by *Ascopus* Marshall, 1951 in the order Coleoptera. Stehlík and Kerzhner (1999) proposed a new name for the genus, *Ascopocoris* Stehlík & Kerzhner, 1999, and placed *Brancuccianabhutanensis* Ahmad & Zaidi, 1986, *Euscopesgestroi* Distant, 1903, and *Antilochuspygmaeus* Distant, 1903 in it. Additionally, Stehlík and Jindra (2006a) established a new subgenus, *Rubriascopus*, with *Antilochuspygmaeus* Distant, 1903 as type species and found a sixth species, *Ascopocorisconstanti* Stehlík & Jindra, 2006. Stehlík and Jindra (2006a) thought that Liu’s (1987) description of *Ascopussinuaticollis* Liu, 1987 was insufficient and suggested it might be *Ascopocorisgestroi* (Distant, 1903), but confirmation of this awaits further study. Stehlík (2007) found that *Brancucciana* is indeed the oldest valid name for this genus-level taxon and placed the above six species in it as new combinations. The seventh species, Brancucciana (Rubriascopus) orientalis Stehlík & Jindra, 2008 is distributed in Philippines and Indonesia (Stehlík and Jindra 2008).

In China, two species of the genus *Brancucciana* have been previously reported. Hsiao (1964) reported B. (Brancucciana) rufa, and Liu (1987) reported B. (Brancucciana) sinuaticollis, both from Yunnan Province. We collected one female and two males at the Nonggang National Nature Reserve, in the Guangxi Zhuang Autonomous Region, southwestern China, and one female from Hainan Island, South China. These specimens are morphologically consistent with Brancucciana (Rubriascopus) orientalis and represent the first report of the species in China. In addition, we reviewed the generic characters according to Hsiao (1964) and Ahmad and Zaidi (1986).

### ﻿Key to the Chinese species of Genus *Brancucciana* Ahmad & Zaidi, 1986

**Table:** 

1	Body generally red; hemelytron red, membrane black; pronotum red; body nearly oval; body with dense and black punctures; legs unicolorous and black	**B. (Rubriascopus) orientalis Stehlík & Jindra, 2008**
–	Body generally reddish brown to black; hemelytron reddish brown to black, anterior margin of corium red to reddish brown; pronotum reddish brown to black, lateral margin red to reddish brown; body almost parallel-sided; body with dense, deep punctures; legs bicolor and mostly yellowish brown	**2**
2	Middle part of lateral margin of pronotum nearly straight; labium and legs nearly uniformly reddish brown	**B. (Brancucciana) rufa (Hsiao, 1964)**
–	Middle part of lateral margin of pronotum inward concave; labium and legs dark brown, apical part of femora and basal part of tibiae red	**B. (Brancucciana) sinuaticollis (Liu, 1987)**

### ﻿ Brancucciana (Rubriascopus) orientalis

Taxon classificationAnimaliaHemipteraPyrrhocoridae

Stehlík & Jindra, 2008

00889B0A-4728-5290-AF86-699D556F7ACA

**Figure 1.**
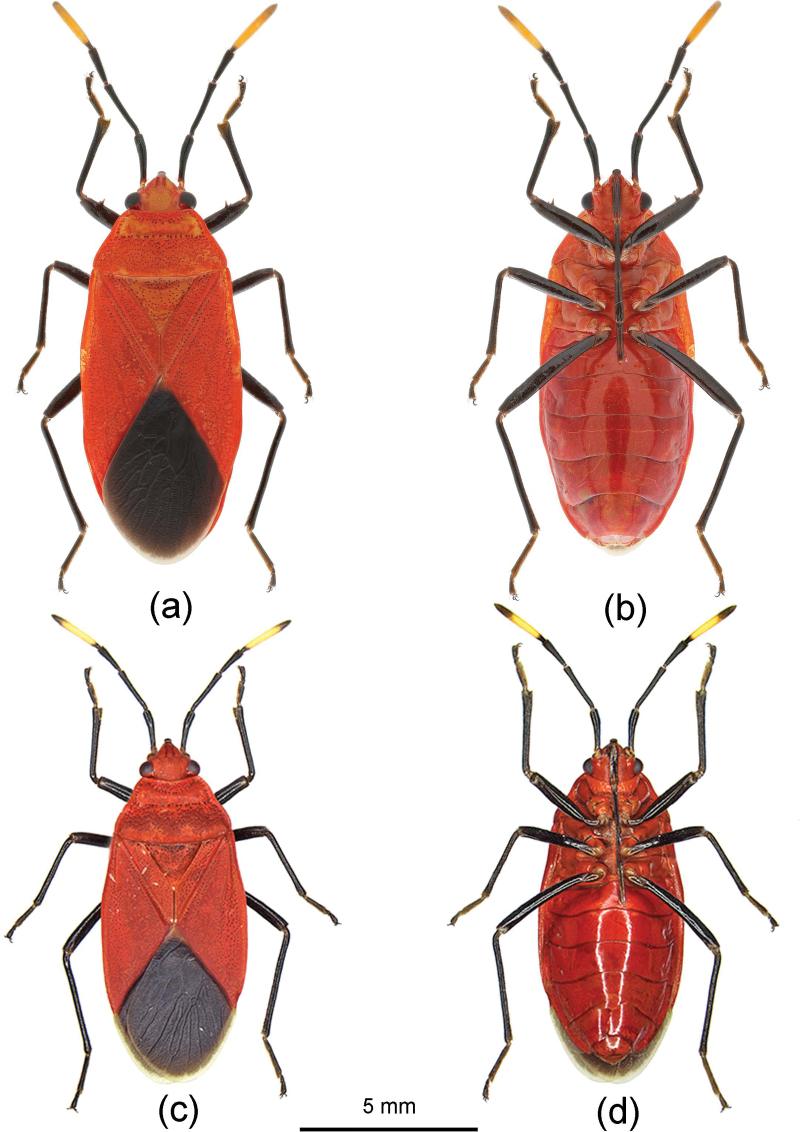
Brancucciana (Rubriascopus) orientalis Stehlík & Jindra, 2008, habitus **a, b** female **c, d** male **a, c** dorsal view **b, d** ventral view.

**Figure 2.**
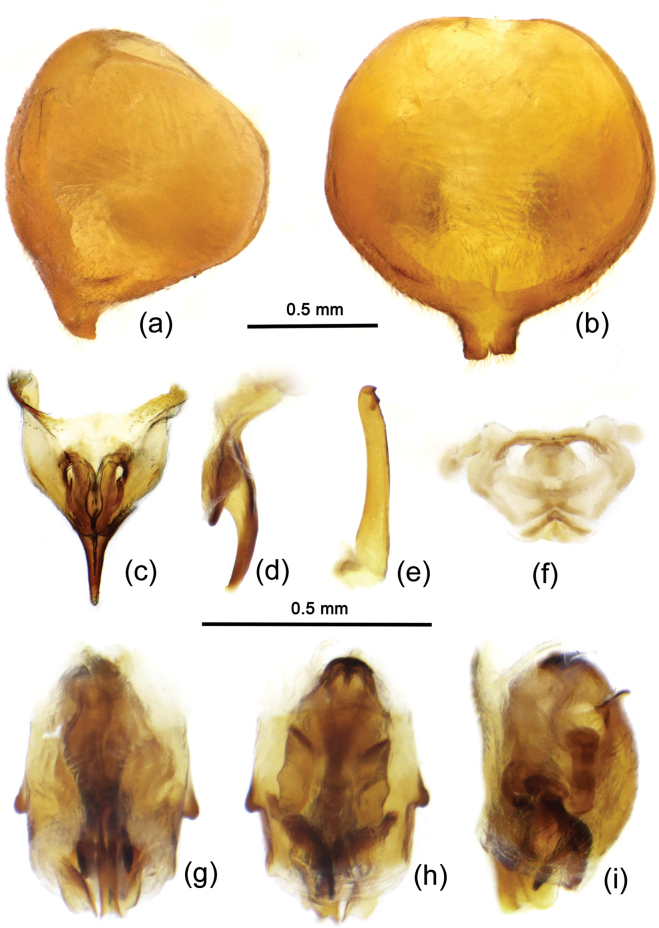
Brancucciana (Rubriascopus) orientalis Stehlík & Jindra, 2008, male external genitalia **a, b** pygophore **c, d** cuplike sclerite of pygophore **e** paramere **f** phallobase **g–i** phallosoma **c, h** dorsal view **a, d, i** lateral view **b, g** ventral view.

Figs 1 , 2 Chinese common name: 东方华红蝽

Brancucciana (Rubriascopus) orientalis Stehlík & Jindra, 2008: 632: Aukema et al. 2013: 402.

#### Diagnosis.

The subgenus Rubriascopus has two species, Brancucciana (Rubriascopus) pygmaeus Distant, 1903 and Brancucciana (Rubriascopus) orientalis Stehlík & Jindra, 2008. In B. (R.) pygmaeus, the legs and antennomeres are greyish yellow according to Stehlík and Jindra (2006a: fig. 5) [vs legs and antennomeres black in B. (R.) orientalis].

The specimens recently collected from Guangxi and Hainan are morphologically mostly consistent with the original description of B. (R.) orientalis according to the description by Stehlík and Jindra (2008). However, based on these Chinese specimens, the body is mostly red, with black markings (vs pale brown with a reddish tinge in the specimen of this species from Indonesia and the Philippines (Stehlík and Jindra 2008). The structure of their male genitalia is consistent with B. (R.) orientalis, therefore we consider the differences in colouration as intraspecific variation.

The male genitalia are redescribed in detail here (Fig. 2). The pygophore is oval, the median pygophore process is armed with a posteriorly produced protrusion, its two lateral margins are parallel and the posterior margin of apical part is nearly straight and medially a little concave (Fig. 2a, b); the ventral rim infolds and falls into genital chamber, and forms a special structural interior process, a cup-like sclerite of pygophore, its apical part acute, as shown in Fig. 2c, d; the paramere is clavate, gradually attenuated, its apical part has a pair of minute denticles (Fig. 2e); the phallosoma is elliptic; the phallobase is shown as in Fig. 2f; the endosoma is shown in Fig. 2g–i, the vesical seminal duct (gonopore) extends to the apical part of the vesica.

#### Measurements

[male (*n* = 1) / female (*n* = 1), in mm]. Body length 9.88 / 11.66; maximal width of abdomen 4.13 / 4.70. Head length 1.30 / 1.70; Head width 1.78 / 1.94; length of synthlipsis 0.97 / 1.30; length of antennal segments I–IV = 1.27 / 1.46, 1.36 / 1.62, 0.91 / 0.97, 1.82 / 1.78; length of labial segments I–IV = 1.13 / 1.16, 1.30 / 1.30, 1.13 / 1.13, 1.13 / 0.97; length of pronotum 3.29 / 3.56; width of pronotum 3.40 / 3.73; length of anterior pronotal lobe 0.81 / 0.81; length of posterior pronotal lobe 1.13 / 1.30; length of scutellum 1.13 / 1.62; length of hemelytron 7.45 / 8.42.

#### Material examined.

2 males, 1 female, China, Guangxi, Longzhou, Nonggang, 2021-VI-14, by light, Ping Zhao & Yingqi Liu leg., deposited in CAU; 1 female, China, Hainan, Dongfang city, Guangyin road, 2023-VI-28, Jianyun Wang leg., deposited in CAU.

#### Distribution.

China [Guangxi (Longzhou), Hainan]; Indonesia, Philippines (Stehlík and Jindra 2008). New record from China

#### Biology.

B. (R.) orientalis demonstrates positive phototaxis when exposed to artificial light sources during nocturnal periods.

### ﻿Genus Dindymus

Taxon classificationAnimaliaHemipteraPyrrhocoridae

Stål, 1861

DF6DFDD0-A1BC-5374-93A7-0B99B988B6F7

Dindymus Stål, 1861: 196; Liu 1981a: 228; Rédei et al. 2009: 23. Type species by subsequent designation (Distant 1903: 110): Dysdercusthoracicus Stål, 1855 (= Pyrrhocorisbicolor Herrich-Schäffer, 1840).

#### Generic character.

Body medium-sized, smooth. Head triangular, vertex bulged, posterior lobe of head quickly constricted into the neck, head not wider than anterior pronotal lobe, apical part of head slightly declined downward; first antennal segment longest, fourth separately longer than second and third segment, second slightly longer than third; eyes sessile, protruded laterally; apical part of labium usually extending beyond base of abdomen, first labial segment slightly thickened. Pronotum trapezoidal, lateral pronotal margins strongly reflexed. Inter-segmental sutures of third to fifth abdominal segments moderately curved laterally.

#### Distribution.

Oriental and Australian region.

#### Remark.

There are six species of the genus *Dindymus* reported from China prior to this study. *Dindymusbrevis* Blöte, 1931 was reported in Taiwan Province (Rédei et al. 2009), *D.medogensis* Liu, 1981 was reported from Xizang Autonomous Region (Liu 1981b), D. (Dindymus) chinensis Stehlík & Jindra, 2006 was described based on materials from Hubei Province (Stehlík and Jindra 2006b), *D.lanius* Stål, 1863, *D.rubiginosus* (Fabricius, 1787), and *D.sanguineus* (Fabricius, 1794) in China are widespread species in continental areas of Oriental region. Herein, we found seventh species from Yunnan Province of China, reported below.

### ﻿Key to the Chinese species of Genus *Dindymus* Stål, 1861

**Table:** 

1	Legs black, apical half of femora and basal 1/4 of tibiae red	**2**
–	Legs completely black	**3**
2	Posterior pronotal lobe red	***D.brevis* Blöte, 1931**
–	Posterior pronotal lobe yellowish-white	***D.albonotum* Zhao & Cao, sp. nov.**
3	First labial segment red; lateral margin of pronotum and costal margin of corium distinctly upturned; membrane of hemelytron without a dark spot in inner corner	**4**
–	First labial segment black, only basal part red; lateral margin of pronotum and costal margin of corium straight, indistinctly upturned; membrane of hemelytron with a dark spot in inner corner	**6**
4	Labium almost extending to middle part of third abdominal sternum, first segment thicker, significantly exceeding beyond anterior margin of pronotum; lateral margin of pronotum and costal margin of corium strongly expanded laterally, almost lamellate	***D.medogensis* Liu, 1981**
–	Labium not extending to middle part of third abdominal sternum, first segment not extending beyond anterior margin of pronotum; lateral margin of pronotum and costal margin of corium only slightly expanded laterally	**5**
5	Body large, male 12.00–14.50 mm, female 15.00–16.50; vertex wider, lateral margin of pronotum and costal margin of corium wider; posterior margins of thoracic pleura pale yellow or reddish, acetabula black; corium and clavus with deeper punctures	***D.lanius* Stål, 1863**
–	Body small, male 11.77–12.31 mm, female 12.85–14.71 mm; vertex wide, lateral margin of pronotum and costal margin of corium wide; posterior margins of thoracic pleura and acetabula milk-white; corium and clavus with shallow punctures	***D.chinensis* Stehlík & Jindra, 2006**
6	Posterior margins of all thoracic pleura milk-white to yellowish white, outer side of coxae with distinct white spot	***D.rubiginosus* (Fabricius, 1787)**
–	Posterior margins of metapleura and posterior acetabula milk-white, outer side of coxae paler	***D.sanguineus* (Fabricius, 1794)**

### ﻿ Dindymus albonotum

Taxon classificationAnimaliaHemipteraPyrrhocoridae

Zhao & Cao sp. nov.

B8F8627A-2BE3-55BA-8492-8BA071F0CE7D

https://zoobank.org/489A818E-C49C-404B-8C65-C2B5C7CFD463

**Figure 3.**
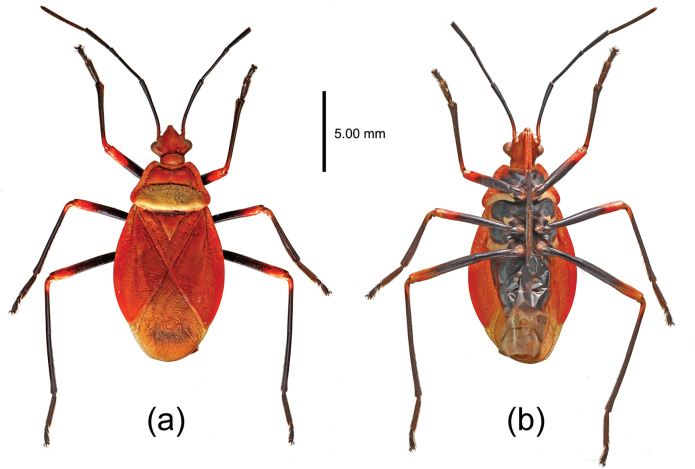
Holotype of *Dindymusalbonotum* Zhao & Cao, sp. nov., female, habitus **a** dorsal view; **b** ventral view.

Fig. 3 Chinese common name: 黄胸光红蝽

#### Diagnosis.

The new species, from Yunnan, China, is similar in body shape and coloration to *Dindymusbrevis* Blöte, 1931, which is distributed in Taiwan, China. However, for *D.albonotum* sp. nov, the posterior lobe of pronotum is milk-white to yellowish white; the fore-wing membrane is golden yellow, semitransparent, and its posterior part has scattered black markings; the abdominal sterna are black, except for the milk-white posterior margin of fifth segment, and sixth and seventh segments (Fig. 3a, b). In *D.brevis*, the pronotum is completely red, the membrane of fore wing is golden yellow, with a large black round spot; the sternum of abdomen is white except the basal part is black, and the apical part is red (Rédei et al. 2009).

The new species also resembles Dindymus (Dindymus) flavinotum Stehlík, 2013 in the following characters: the posterior pronotal lobe is milk-white, and the membrane of fore wing is golden yellow and with fewer black markings; the legs are black, with apices of the femora and bases of the tibiae red; the second to fifth abdominal sternites are black; the sixth and seventh are white. In *D.flavinotum*, the legs are completely black, and the abdominal sternites are red (except black basal parts) (Stehlík 2013).

#### Type species.

***Holotype***, female, China, Yunnan, Puer, 2022-VI, Zhang Guirong leg., deposited in CAU.

#### Description.

***Coloration*.** Body red with black and milk-white markings. Antennae black, basal part of first segment red; labium blackish brown, first segment red; posterior pronotal lobe milk white; pleura and sterna of thorax black, posterior margin of pleura and posterior margin of acetabula milk white; leg black, apical part of femora and basal part of tibiae red; abdomen black, posterior margin of fifth sternum of abdomen, sixth and seventh abdominal sternites milk white.

***Structure*.** Body oval. Pronotum and fore wing widened transversely. Head length subequal to width, eyes laterally protruded; anterior and posterior lobe of pronotum gibbose; anterior margin of pronotum not wider than head, and subequal to 1/2 of distance between lateral pronotal angles. Posterior margin of anterior pronotal lobe convex anteriorly; posterior pronotal lobe sparsely punctured. Costal margin of corium laterally dilated and smooth, corium (except costal margin) and clavus densely punctured.

#### Measurements

[female (*n* = 1), in mm]. Body length 14.78; maximal width of abdomen 7.13. Head length 2.78; head width 2.61; length of synthlipsis 1.39; length of antennal segments I–IV = 4.35, 2.61, 1.91, 3.48; length of labial segments I–IV = 2.52, 2.52, 2.09, 1.22; length of pronotum 2.86; width of pronotum 4.96; length of anterior pronotal lobe 1.22; length of posterior pronotal lobe 1.74; length of scutellum 2.73 length of hemelytron 12.17.

#### Etymology.

The specific name alludes to the yellow posterior lobe of pronotum of the new species. The Latin noun *albonotum* means “yellowish-white thorax”.

#### Distribution.

China [Yunnan (Puer)].

#### Biology.

The specimen was collected from the forest near Wanmu tea garden, in Puer, Yunnan, China.

### ﻿Genus Euscopus

Taxon classificationAnimaliaHemipteraPyrrhocoridae

Stål, 1870

0A55F02B-5DD8-564B-815E-2898AADB7721

Euscopus Stål, 1870: 102: Distant 1903: 105; Liu 1981a: 231. Type species by monotypy: Euscopusrufipes Stål, 1870.

#### Generic character.

Body oblique and covered with dense, short setae. Head length approximately equal to width, its apical part downward declining, slightly bulging at top, head width approximately equal to width of anterior pronotal lobe; labium extending to or over coxae of the middle leg, first segment not longer than head; antennae slightly thicker and shorter, first segment longest, second to fourth segments approximately equal in length; pronotum wider than long, lateral margin slightly upward upturned, transversal constriction obviously continuous. Costal margin of corium of the fore wing nearly straight; fore femora slightly thickened, subapical part of the ventral surface with two or three small spines; intersegmental sulcus of the fourth and fifth segments of abdominal sterna obviously bent forward, not straight to its lateral margin.

#### Remark.

A total of 16 species are currently recognized worldwide (Zhao et al. 2022), with four species reported in China. A new record species is reported from China in this paper.

#### Distribution.

Oriental region.

### ﻿Key to the Chinese species in genus *Euscopus* Stål, 1870

**Table:** 

1	Corium of fore wing black, anterior margin red	**2**
–	Corium of fore wing red, middle part with a large black spot, apical angle black	**4**
2	Posterior margin of pronotum red	**3**
–	Posterior margin of pronotum not red	***E.robustus* Stehlík, 2005**
3	A smooth white point near the centre of the apical edge of the corium	***E.distinguendus* Blöte, 1933**
–	Apical part of the corium without with white spot	***E.fuscus* Hsiao, 1964**
4	Apical angle of corium with a small black spot on inner side; membrane of fore wing blackish brown, its outer and inner margin light brown; abdomen ventrally completely black	***E.rufipes* Stål, 1870**
–	Apical angle of corium with larger black markings on inner side; membrane of fore wing greyish brown; abdomen ventrally black with median longitudinal part red	***E.chinensis* Blöte, 1932**

### ﻿ Euscopus robustus

Taxon classificationAnimaliaHemipteraPyrrhocoridae

Stehlík, 2005

8D610684-BB8D-5157-8C0E-64BB57CAB052

**Figure 4.**
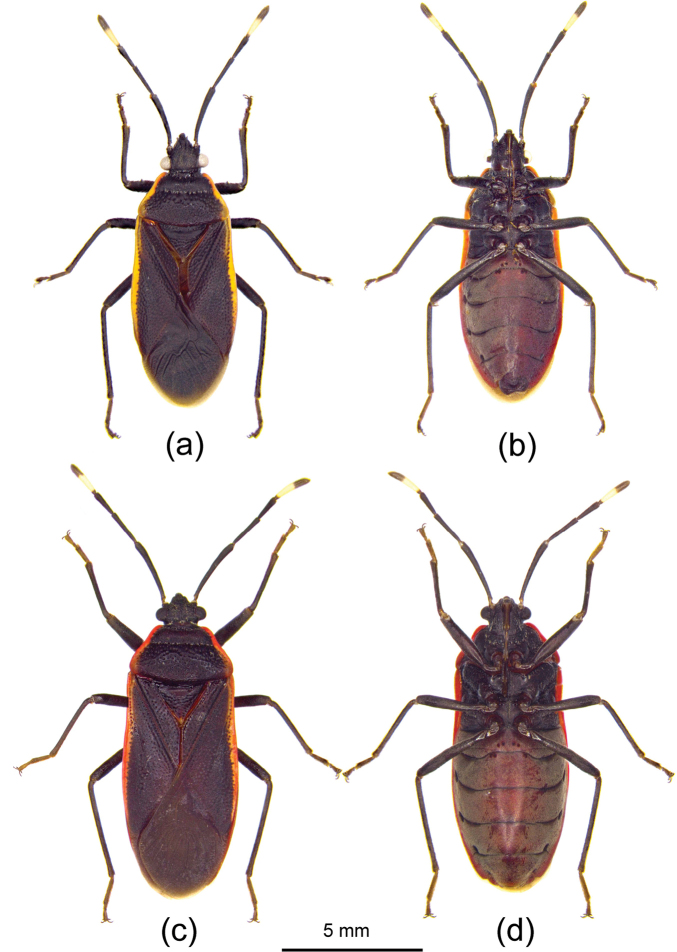
*Euscopusrobustus* Stehlík, 2005, habitus **a, b** male **c, d** female **a, c** dorsal view **b, d** ventral view.

**Figure 5.**
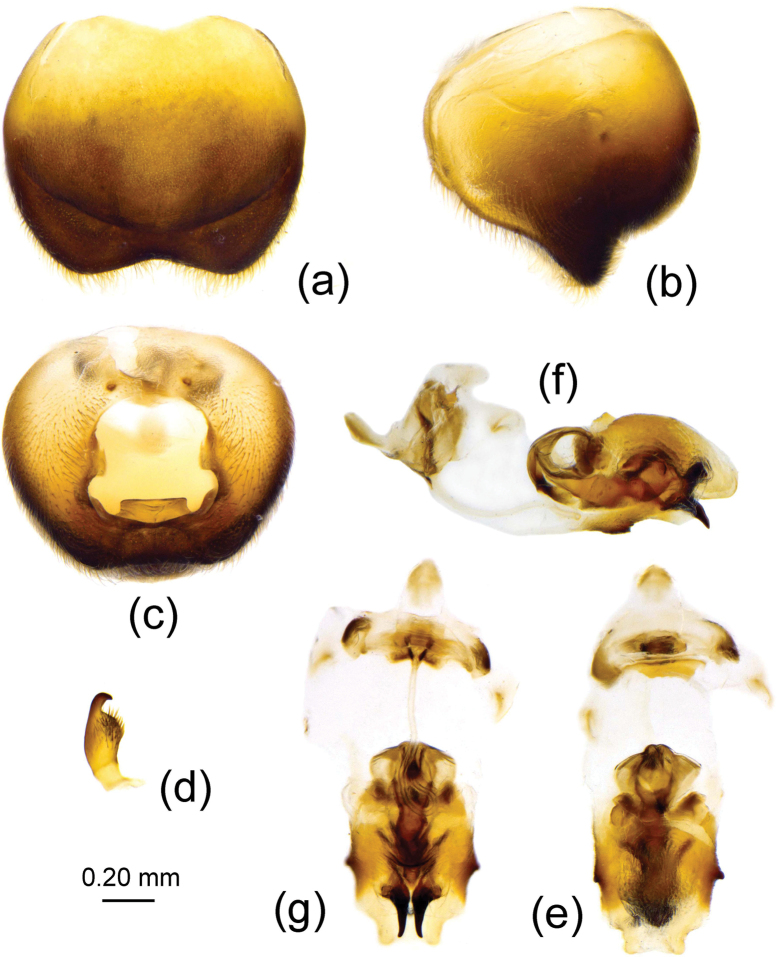
*Euscopusrobustus* Stehlík, 2005, male external genitalia **a–c** pygophore **d** paramere **e–g** phallus **a, e** dorsal view **b, f** lateral view **g** ventral view **c** caudal view.

Figs 4 , 5 Chinese common name: 黑锐红蝽

Euscopus robustus Stehlík, 2005: 157.

#### Diagnosis.

The species is similar to *Euscopusfuscus* Hsiao, 1964 in body shape and coloration. However, the species reported here lacks the yellow to red posterior margin on the pronotum, and the body is nearly parallel-sided (in *E.fuscus*, the posterior margin of pronotum is yellowish brown, and the body is oblong-elliptical).

#### Redescription.

***Coloration*.** Body dorsally black, ventrally reddish brown. Head, thorax, scutellum, and legs black; lateral margin of pronotum, costal margin of corium yellow to red; antennae black, basal half of fourth white (Fig. 4).

***Structure*.** Body clothed with procumbent short setae. Posterior pronotal lobe, clavus, corium, rim of callus of anterior pronotal lobe, scutellum punctate. Body oblong, nearly parallel-sided (Fig. 4). Head subangular, compound eyes laterally produced, apical part of head forward and downward sloping; frons bulged; clypeus thickened and longer than paraclypei; first antennal segment longest, second sub-equal to fourth in length, third shortest; first to third labial segments nearly equal in length, fourth shortest, first and fourth thickened. Pronotum slightly gibbous, lateral pronotal margin moderately wide and reflexed; anterior pronotal lobe subequal to in length posterior lobe. Profemora ventrally in apical half with several minute denticles, middle part with a large spine. Third and fourth abdominal sternites with four round black spots, and two sides of each abdominal sternum with two black spots.

#### Male genitalia.

Pygophore gibbous, posterior margin concaved medially, posterior rim infolding and distinctly sloping into genital chamber, and forming an internal cuplike sclerite of pygophore, its apical margin straight (Fig. 5a–c); parameres short and thick, middle part with many oblique setae, apical part with a hook-shaped process (Fig. 5d); phallosoma shown in Fig. 5e–g, a pair of dorsal conjunctival appendages long horn-shaped.

#### Measurements

[male (*n* = 1) / female (*n* = 1), in mm]. Body length 9.56 / 11.04; maximal width of abdomen 3.74 / 4.00. Head length 1.22 / 1.48; Head width 1.74 / 1.74; length of synthlipsis 0.87 / 0.96; length of antennal segments I–IV= 2.00 / 2.26, 1.22 / 1.30, 0.87 / 0.87, 1.30/1.30; length of labial segments I–IV = 0.78 / 0.78, 0.78 / 0.87, 0.70 / 0.87, 0.43 / 0.43; length of pronotum 1.74 / 2.26; width of pronotum 3.22 / 3.65; length of anterior pronotal lobe 0.70 / 0.70; length of posterior pronotal lobe 1.04 / 1.39; length of scutellum 1.30 / 1.22; length of hemelytron 6.96 / 8.00.

#### Material examined.

1 male, 1 female, China, Guangxi, Nonggang National Nature Reserve, Ningming, Huashan, Ping Zhao, Zhuo Chen & Yingqi Liu leg., 2022-VII-11, collected by using a light trap, deposited in CAU.

#### Distribution.

China [Guangxi(Nonggang)]; Laos. New record from China

#### Biology.

*Euscopusrobustus* exhibits positive phototaxis when exposed to artificial light sources during nocturnal periods.

## Supplementary Material

XML Treatment for Brancucciana

XML Treatment for Brancucciana (Rubriascopus) orientalis

XML Treatment for Dindymus

XML Treatment for Dindymus albonotum

XML Treatment for Euscopus

XML Treatment for Euscopus robustus

## ﻿Additional information

### Author ORCIDs

Ping Zhao https://orcid.org/0000-0001-9315-2756

Minmin Ou https://orcid.org/0009-0001-2623-293X

Liangming Cao https://orcid.org/0000-0002-6581-6719

Jianyun Wang https://orcid.org/0000-0002-8466-3252

### Data availability

All of the data that support the findings of this study are available in the main text.
